# Prospects of Synthesized Magnetic TiO_2_-Based Membranes for Wastewater Treatment: A Review

**DOI:** 10.3390/ma14133524

**Published:** 2021-06-24

**Authors:** E. Kweinor Tetteh, S. Rathilal, D. Asante-Sackey, M. Noro Chollom

**Affiliations:** Green Engineering and Sustainability Research Group, Department of Chemical Engineering, Faculty of Engineering and the Built Environment, Steve Biko Campus, Durban University of Technology, Durban 4001, South Africa; rathilals@dut.ac.za (S.R.); ingsackey@gmail.com (D.A.-S.); mnchollom@gmail.com (M.N.C.)

**Keywords:** advanced oxidation process, titanium dioxide (TiO_2_), photocatalysis, magnetic TiO_2_, membranes, wastewater treatment

## Abstract

Global accessibility to clean water has stressed the need to develop advanced technologies for the removal of toxic organic and inorganic pollutants and pathogens from wastewater to meet stringent discharge water quality limits. Conventionally, the high separation efficiencies, relative low costs, small footprint, and ease of operation associated with integrated photocatalytic-membrane (IPM) technologies are gaining an all-inclusive attention. Conversely, photocatalysis and membrane technologies face some degree of setbacks, which limit their worldwide application in wastewater settings for the treatment of emerging contaminants. Therefore, this review elucidated titanium dioxide (TiO_2_), based on its unique properties (low cost, non-toxicity, biocompatibility, and high chemical stability), to have great potential in engineering photocatalytic-based membranes for reclamation of wastewater for re-use. The environmental pathway of TiO_2_ nanoparticles, membranes and configuration types, modification process, characteristics, and applications of IPMs in water settings are discussed. Future research and prospects of magnetized TiO_2_-based membrane technology is highlighted as a viable water purification technology to mitigate fouling in the membrane process and photocatalyst recoverability. In addition, exploring life cycle assessment research would also aid in utilizing the concept and pressing for large-scale application of this technology.

## 1. Introduction

In a growing economy, clean and drinkable water, free from toxic chemicals, carcinogenic substances and harmful microorganisms are essential for human health and sustainability [1,2]. However, the fast population growth associated with urbanization, irrigated agriculture and industrialization have driven the environmental concern of wastewater and demand for fresh water globally [3,4]. This opioid epidemic, be it due to insufficient accessibility to clean water, poor water quality or waterborne diseases, has seriously threatened human lives in many underdeveloped and developing countries [4,5,6]. The result has been an upsurge of stringent environmental standards to provide clean water with high quality and sustainable waste management facilities [4,5,6,7]. In addition, conventional technologies (coagulation, flocculation, biological) are limited when it comes to complete decontamination of water containing emerging contaminants (hormones, persistent organic pollutants, antibiotics, pharmaceuticals, heavy metals, nano plastics, etc.) [6,8,9,10], and to avoid secondary pollution problems an advanced treatment technology is required. It is therefore valuable to explore the development of advanced wastewater treatment technologies to effectively purify water in a more ecofriendly and economical routine.

In recent years, advances in nanoengineering and nanotechnology to develop nanosorbents, nanocatalysts, bioactive nanoparticles, and filtration systems coupled with nanoparticles have been seen to be very promising in wastewater treatment settings [1,9,11,12,13,14,15]. Most of the nanotechnology-based materials can exhibit distinctive physical and chemical properties at a nanoscale (1–100 nm) as compared to their bulk complements [9,12]. Intensive research devoted to understanding the fundamental mechanism of nano-based materials has revealed that semiconductor photocatalysts, such as TiO_2_, possess high surface-to-volume ratios, chemical stability, hydrophilicity and high photo-reactivity with antireflection and self-cleaning abilities [13,16,17]. Semiconductor photocatalysis is driven by energy sources (UV light, ultrasonic, heat or sunlight) to activate metal oxides (TiO_2_, ZnO, and Fenton reagent) in order to generate radical species (OH−, H+) in the occurrence of wet or air oxidation states like ozone or hydrogen peroxide [10,18,19,20,21].

Heterogeneous photocatalysts and semiconductors assisted with UV light for photodegradation of organic pollutants in water and wastewater settings have been reported by many researchers [9,11,16,22,23]. These include BiVO_4_, TiO_2_ Fe–ZnIn_2_S_4_, WO_3_, BiOBr, BiFeO_3_, Fe_2_O_3_, CuS, and ZnO [11,17,24]. Among these photocatalysts and nanoparticles, titanium dioxide (TiO_2_), being cheap, commercially available, nontoxic and chemically stable, has been the most extensively researched in water settings [25,26,27]. Aside from water and wastewater treatment applications, the TiO_2_ photocatalyst has been widely used in air purification and other biological applications [25,28,29,30,31,32,33]. Notwithstanding, there are several setbacks associated with TiO_2_ industrial applications at a large scale via the advanced oxidation process (AOP) [11,18,25,34,35]. Some of these include high cost of energy resources and chemicals as oxidants and their potential to handle large amounts of wastewater with high organic strengths [11,34,35]. The separation, recovery, and re-use of TiO_2_ nanoparticles is also a major constraint to its industrial applications [34,36,37].

In general, most chemicals or nanoparticles used in conventional systems end up converting contaminants from one form to another [11,15]. For instance, generation of biosolids or sludge becomes the new pollutant, which requires further treatment before discharge. Additionally, in the application of TiO_2_ photocatalysis, the low rate of electrostatic interaction to oxygen and high rate of electron-hole recombination significantly slows its adsorption of organic contaminants onto its surface [11,38]. Yet, there are still some research gaps associated with TiO_2_ nanoparticle applications, which include low quantum efficiency due to inefficient visible light harvesting, acceptable photoreactor design, recovery, re-use, scale-up, etc. [25]. Thus, in conjunction with unaddressed concerns of emerging contaminants [1], the search for ecofriendly techniques capable of degrading pollutants rather than simply converting them from one form to another becomes essential. Consequentially, many researchers have pursued to develop new heterogeneous photocatalysts with a suitable crystal structure, high specific surface area, and easy separation and re-use capabilities [18,26,30,39]. This includes synthesizing metal/TiO_2_ nanocomposites as an efficient way to improve TiO_2_ photocatalytic efficiency by enhancing its electron-hole separation [18,30,40]. In addition, the embedding of Fe_3_O_4_ has been proven to be very useful in order to provide TiO_2_ with magnetic separation ability [41,42,43].

### Research Approach

**Figure 1:**
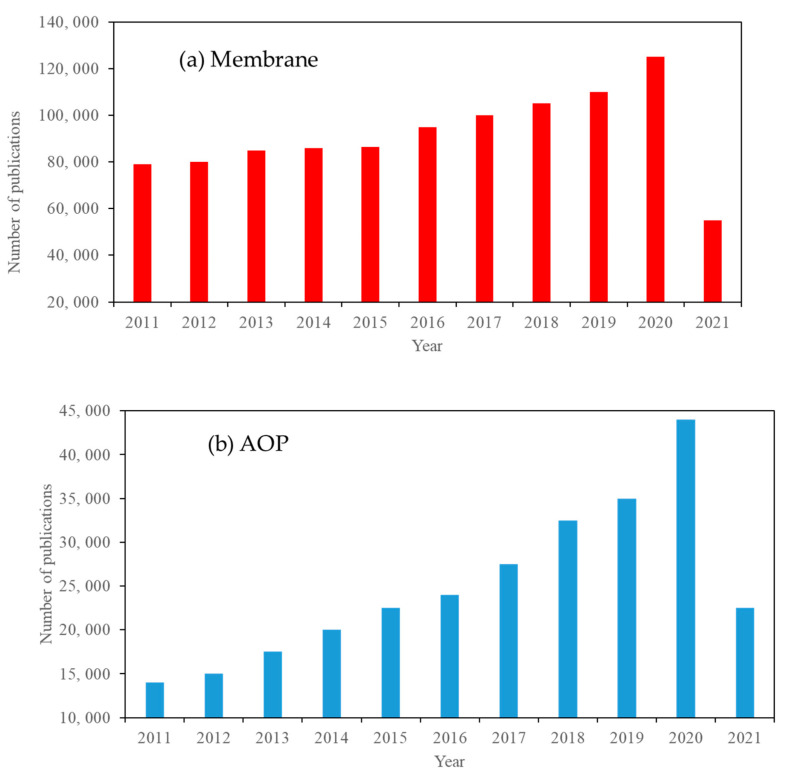

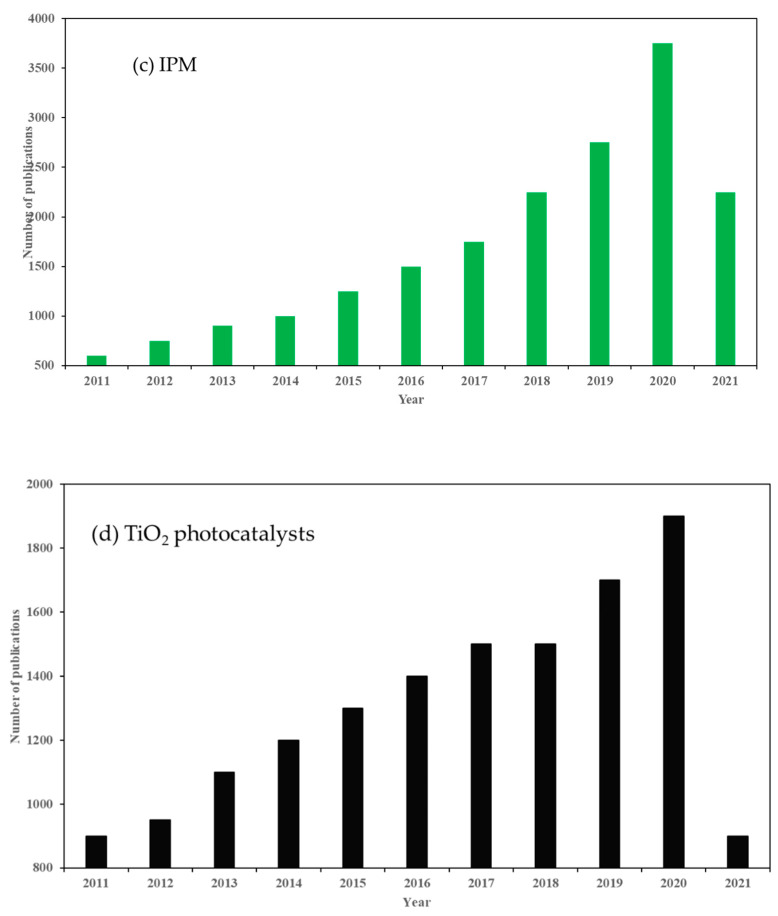
Publication trend of (**a**) membranes, (**b**) advanced oxidation processes (AOP), (**c**) integrated photocatalytic processes (IPM), and (**d**) TiO_2_ photocatalysts, from 2011–2021 (Web of Science database, 20 April 2021).

Conventionally, post-treatment of wastewater with membrane filtration or TiO_2_ immobilization via AOP has been applied and reported by many researchers. These processes, however, face problems such as membrane fouling and loss of effective catalysts [30,44,45]. Although substantial progress has been made in developing membrane technologies at a large scale, much work is expected for TiO_2_ photocatalysis to become a commercialized technology in wastewater treatment settings. Therefore, in this review the basics of photocatalysis are discussed with TiO_2_ nanoparticles and recombination with membrane technology as well as the mechanisms involved to prioritize their usage in the water and wastewater treatment sector [46,47,48]. This was carried out via a desktop approach, where a search of literature with keywords such as membrane, TiO_2_ photocatalyst, advanced oxidation process (AOP) and integrated photocatalytic membrane (IPM) was conducted utilizing Google scholar, Web of Science, PubMed, and the ScienceDirect database, as shown in Figure 1. The data obtained from 2011–2021 were refined based on the following categories: subscribed journals, article type, publication title, subject area and access type (open access or achieve). Even though the progression of membrane technology (Figure 1a) as of 20 April 2021 seems to be low (<60,000), its publication supersedes AOP (Figure 1b; <25,000), followed by IPM (Figure 1c; <2500), and then TiO_2_ photocatalysis (Figure 1d; <1000). Apparently, the knowledge of IPM is still limited and therefore should be given attention.

## 2. Advanced Oxidation Process (AOP)

Advanced oxidation processes (AOPs) have attracted a great deal of attention of stakeholders in the water and wastewater treatment sector. Photocatalytic degradation technology, being one of the AOPs, involves the use of a semiconductor photocatalyst and photogeneration of highly oxidative hydroxyl radicals [22,34]. In addition, the chemical reactions in AOPs caused by the absorption or desorption of photocatalysts remains unchanged in both photocatalysis and photoelectrochemical splitting of water using titania [19,22,49]. Furthermore, AOPs differ from conventional chemical and biological wastewater treatment systems as their produced hydroxyl radicals are potent oxidants used to degrade toxic and recalcitrant contaminants into simple and harmless inorganic molecules without creating a secondary excess [46,50,51]. Additionally, AOPs are very robust so as to enhance precipitations and elimination of heavy metals as metal hydroxides, and subsequently, based on the irradiation period, can lead to total mineralization [19,34,52]. AOPs are classified into two categories, namely homogeneous and heterogeneous systems, that can be carried out with or without UV radiation. Table 1 presents some of the reported AOPs commonly used in wastewater treatment settings. The thermal Fenton reaction process is a homogeneous process that involves Fe^2+^ reacting with H_2_O_2_. The aforementioned phase (Fe^2+/^Fe^3+,^ H_2_O_2_, UV-Vis) is commonly termed as a photo-Fenton reaction [19]. Heterogeneous photocatalysis involves a photoinduced reaction accelerated by the presence of TiO_2_ photocatalysts [53,54]. This process stands out among the heterogeneous AOPs because UV irradiation of TiO_2_ creates hydroxyl radicals. This facilitates the oxidation and more efficient mineralization of organic material present in the effluent when it is photocatalyzed [55]. In contrast with other AOPs and biological treatments, TiO_2_ photocatalysis offers significant advantages [19,46]. Thus, the TiO_2_/UV system can handle contaminants in both the gas and solution phases. Furthermore, TiO_2_ is inexpensive, practically insoluble in water, and biologically and chemically inert [19,22,46].

### 2.1. TiO_2_ Photocatalyst

TiO_2_ is the most widely used photocatalyst because of its strong physical and chemical stability, insolubility in water, resistance to acids, inertness to most chemicals and long-term photostability [25,40,47]. TiO_2_ exhibits three main crystal structures such as rutile (stable), anatase (metastable), and brookite structures (Table 2).

The tetragonal structure consists of both rutile and anatase, while brookite has a rhombohedral structure [48,49,50]. In addition to their shape and nanoscale or size, the unique physicochemical properties of TiO_2_ crystals are a result of their intrinsic electronic structure and crystal structures [17,25,51]. Anatase has been found to be more active than rutile, but both forms of TiO_2_ have been reported to be photoactive. Additionally, a mixture of anatase and rutile for photocatalysis has been found to exhibit synergy due to the photoinduced interfacial electrons of anatase that can be transformed into rutile [27,39].

**Figure 2:**
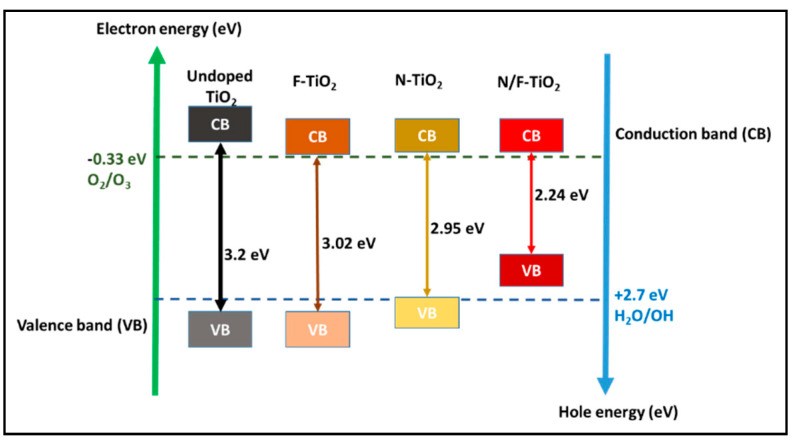
Schematic diagram of TiO_2_ with different valence and conduction band levels; adapted from [49,53].

Rutile and anatase, with 3.0 eV and 3.2 eV band gaps, respectively, are the most common polymorphs of TiO_2_ [49,50]. In Figure 2, the large TiO_2_ energy band means that higher UV light energy is required to excite electrons to create hydroxyl radicals, which is the key to the photodegradation of contaminants in the presence of oxygen [20,27,52].

The crystalline phase of anatase is desired because of its maximum efficiency under UV irradiation [20]. The photogenerated electrons form superoxide radicals in the rutile conduction band and the holes in the anatase valence band play a significant role in oxidation reactions. In addition, the 3.2 eV anatase band gap (Figure 2) corresponding to the light wavelength of 388 nm means that only UV light will activate TiO_2_. This limits the use of sunlight and greener energy for the activation of TiO_2_, as only around 5% of sunlight is formed by UV radiation. Thus, during the photo activation step, TiO_2_ undergoes a rapid recombination of electrons and holes, which then reduces photoactivity. Hence, by doping the TiO_2_ with inert supports, the electron–hole recombination and wide band gap can be addressed while increasing the TiO_2_ nanoparticle size [20,25].

Furthermore, in photocatalytic activities, TiO_2_ with a valence band (VB) and conduction band (CB) levels of +2.9 and −0.33 eV can result in a band gap energy of 3.2 eV. As shown in Figure 2, the TiO_2_ VB and CB levels are more positive and negative than the distinctive redox potential of O_2_/H_2_O (1.23 eV). The presence of Ti^3+^ defects beneath the CB surface causes the band gap reduction in F-doped TiO_2_ (3.02 eV), while in N-doped TiO_2_ (2.95 eV) the mid-band states are created as the N species fill voids as impurities above the VB. However, there is a doping-induced effect and a transition in the VB tail; co-doping N and F into TiO_2_ results in the greatest band gap reduction, from 3.2 eV to 2.24 eV (Figure 2).

#### 2.1.1. TiO_2_ Modification Supports

In wastewater treatment settings, TiO_2_ photocatalysis in practical applications is very limited due to low quantum efficiency under visible radiations as well as difficulties of being separated in suspensions [18,55,56]. To ensure that TiO_2_ will work optimally without any complexity, modification becomes essential [6,54]. Filtration following photocatalytic activities is very costly and, therefore, catalyst supports can be explored. Some of the supporting materials of TiO_2_ reported include glass beads, fiber, glass, silica and zeolite mater [6,20,25,53,57]. Consequently, there are diverse techniques used to modify TiO_2_, where doping of pure TiO_2_ with either anions or cations remain one of the most common methods for enhancing its sensitivity to visible light and lowering the rate of charge carrier recombination [20,39,53,54]. Furthermore, photocatalysts synthesized via chemical doping or ion implantation with metals and nonmetals result in a narrowing of the TiO_2_ band gap as well as the crystal lattice or structure [20,25,39]. Therefore, in selecting these metals and nonmetals, their sensitivity should be considered.

- Cationic doping of TiO_2_

To dope TiO_2_, cations like rare earth metals, noble metals, weak metals and transition metals have been used [26]. In addition, when a metallic ion is doped with TiO_2_, the light absorption range expands, whereas the redox potential of the photogenerated radicals increases [58,59,60]. This generally results in the transfer of photoexcited electrons from the TiO_2_ conduction band to metal particles deposited on the TiO_2_ surface. Cationic doping can also decrease the probability of electron–hole recombination, leading to an effective separation of the charge and thus improved photocatalytic activity [61]. However, the nature and concentration of the dopant inducing the charge surface together with the corrosion mechanism can affect the photocatalytic property of the materials [20,40]. Thus, cationic dopants have a distinctive impact on the catalyst lattice. Some of these metal dopants (Ag, Fe, Mn, Ni, Cu, Pt, Rh, and Pd) act as free electrons, which facilitates the conjugation of the photogenerated carriers [26]. Conversely, metal-doped materials have been reported to be thermodynamically unstable, which can be captured on the TiO_2_ band gap at the d-level and can subsequently shift the redox potential [26,30,62,63]. However, by removing the electron hole, TiO_2_ doped with metallic cations with lower oxidation states can maintain electron stability [30]. In addition, TiO_2_ doped with metallic cations with higher oxidation states, by adding electrons to the already empty conduction band, can also enhance the electron stability [20,40].

- Anionic doping of TiO_2_

Doping TiO_2_ with anionic nonmetals, including carbon, sulfur, nitrogen, and iodine, has been found to be more efficient in ensuring higher photocatalytic activity under visible light [26]. Anionic dopants result in p-state substitution in TiO_2_ by altering the atomic level in the valance band and subsequent shift of the conduction band. For instance, incorporation of carbon into TiO_2_ contributes to the formation of carbonaceous species on the surface, which increases the visible light absorption [18,58,60]. Furthermore, the presence of more reactive sites, which facilitate the adsorption of more target contaminants, is another explanation for the increase in photocatalytic activity of C-doped TiO_2_ due to its higher surface area [26]. Nitrogen doping in TiO_2_ adjusts the stiffness, elastic modulus, refractive index, electrical conductivity, and photocatalytic behavior of TiO_2_ under visible light absorption activity [18].

Subsequently, silica is reported to have higher surface area, which provides the effective adsorption and catalyst support structure for heterogeneous photocatalysis. According to Ghosh and Das [26], silica modified TiO_2_ is more effective as a photocatalyst than TiO_2_ alone. Thus, doping TiO_2_ with silica can enhance and stabilize both the external and internal surfaces of the catalyst within the silica-based matrix, where contaminants are adsorbed. Zeolite is also found to play an important role in charge and electron transfer during the photocatalytic reaction [20,34]. Due to uniform pores and straight paths, zeolites have been found to be good photocatalyst supports. The zeolite acts as a supporting material for the homogeneous dispersion of TiO_2_ particles on its surface, which improves TiO_2_-based zeolite composites. Activated carbon (AC), due to its pore structure, also has benefits as a photocatalyst support for promoting the photocatalytic process [25,53,64]. AC is commonly used as a supporting material to extract organic and inorganic pollutants from water and wastewater. Contrariwise, the surface chemistry of AC carbon may obstruct effective coating of TiO_2_ photocatalysts [64]. However, the application of AC–TiO_2_ photocatalysts in wastewater treatment settings comes with a cost and, therefore, necessitates the use of a reactor to effectively expose the photocatalytic surface to light photons [2,53,64].

- Other supporting metallic oxides

Coupling TiO_2_ with other semiconductors to increase its photocatalytic activity is another way to produce photoinduced electron–hole pairs [26]. A good match between the conduction and valence bands of two semiconductors ensures a potential charge carrier movement from one to the other. Several studies have indicated that TiO_2_ photocatalytic activity can be enhanced by the presence of metal oxides like CdS, ZnO, ZrO_2_, Cu_2_O, CeO_2,_ SnO_2_, WO_3_, and Fe_3_O_4_ [17,18,26,62,65,66]. Among them, Fe_3_O_4_ has been studied extensively due to its ability to facilitate magnetic separation of the nanocomposite from the aqueous medium [39,67]. Ahangar et al. [60] prepared a Fe_3_O_4_-embedded SiO_2_ nanocomposite and compared its magnetic properties with pure Fe_3_O_4_. It was found that the Fe_3_O_4_ nanoparticles embedded in SiO_2_ have a much higher photocatalytic efficiency. Furthermore, the photocatalytic performance of the resulting nanocomposite was nearly identical. As a result, the core–shell framework is an efficient way to incorporate the desirable properties of two or more nanomaterials into a single nanocomposite. However, adding semiconductor dopants can alter the surface properties of TiO_2_ catalysts, such as surface area and surface acidity [16,41]. Wang et al. [68] used a hydrothermal method followed by co-precipitation to develop anatase-structured TiO_2_ nanoparticles (Fe/TiO_2_) with elongated morphological characteristics.

#### 2.1.2. Mechanism of TiO_2_ Photocatalysis

**Figure 3:**
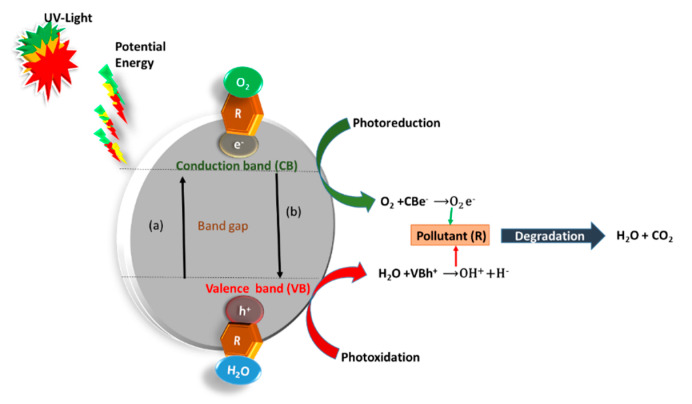
Schematic presentation of the photocatalysis mechanism; adapted from [26].

The heterogeneous photocatalysis mechanism involves the ability of semiconductors to produce charge carriers under light irradiation, preceded by the production of free radicals such as OH^−^, which contributes to further reactions, eventually forming CO_2_ and H_2_O [32]. Excitation, bulk diffusion, light-induced surface transfer, and photon absorption with a potential energy greater than the band gap are all part of the basic photocatalytic activation mechanism [30,47]. As shown in Figure 3, when UV light with an energy greater than the band gap energy of TiO_2_ is irradiated, electrons in the valance band are excited to the conductance band, which leaves positive charge holes (h^+^) in the valance band.

The electrons extracted from the VB are passed to the CB, which then react with the absorbed oxygen (*O*_2_) on the *TiO*_2_ surface. As a result, a positive area (*h*^+^) forms in the *VB* holes and causes free electrons (*e*^−^) to form in the *CB*. Simultaneously, the holes (*h*^+^) react with water molecules (*H*_2_*O*) adsorbed on the *TiO*_2_ surface to create hydroxyl radicals (*OH*), as shown in Figure 3 and Equations (1)–(4). Furthermore, the CB electron reduces oxygen to superoxide ions (*O*_2_^•−^). This reaction prevents the *e*^−^/*h*^+^ from recombining, which happens when other electron acceptors such as pollutants are absent [30,40]. Organic pollutants (*R*) in wastewater can be oxidized to carbon dioxide ($CO_{2})$ and water $\left(H_{2}O\right)$ by oxygen reactive radicals.

(1) $$TiO_{2}\overset{hv}{\to }TiO_{2}+e^{-}\left(CB\right)+h^{+}\left(VB\right)\;$$

(2) $$TiO_{2}\left(h^{+}\right)+OH^{-}\to TiO_{2}+•OH\;$$

(3) $$\;\;O_{2}+e^{-}\to O_{2}{}^{•-}\;\;\left(superoxide\;radicals\right)$$

(4) $$\;\;O_{2}{}^{•-}+R\;\;\to intermediates\;\to \;\;H_{2}O+CO_{2}$$

### 2.2. Operational Parameters in Photocatalysis

The photocatalytic system’s oxidation rates and semiconductor catalyst performance coupled with a photoreactor are all highly dependent on several operational parameters that regulate the kinetics [18,26,69]. Outstanding studies of the different parameters affecting the photocatalysis process, including pH, catalyst loading, amount of oxygen, contaminant loading, light intensity and duration of light irradiation, preparation method of the catalyst, its calcination temperature, and amount and type of dopant, have been carried out and reported in the literature [18,25,26,51,53,70,71]. In comparison to immobilized films, photocatalysts mostly take the form of suspended powder (slurries), giving higher efficiency [25]. However, separating photocatalysts from treated water in this form is difficult, implying that a recovery step for photocatalyst re-use is required [72,73,74]. Photocatalysts based on TiO_2_ with a large surface area have a high photocatalytic efficiency, so nano-sized TiO_2_ particles have been used in most studies [25,74].

- pH

pH affects the surface charge or isoelectric point of the photocatalyst directly as well as the catalyst particles, size of catalyst aggregates, and the positions of conductance and valence bands [75,76]. The point of zero charge (PZC) of TiO_2_ is the main parameter used to study the effect of pH in photocatalyst reactions. PZC is a condition where the surface charge of TiO_2_ is zero or neutral in the range of 4.5–7.0 [69,75]. The catalyst surface will be negatively charged and repel the anionic compounds in water, which can affect the charge densities [75]. At pH = PZC, the neutral surface charge of the catalyst particles will not be able to produce the interactive rejection necessary for the separation of the solid liquid. When this happens, the catalyst becomes larger, thereby leading to the sedimentation of the catalyst. Since this can induce the aggregation of the catalyst, optimization becomes necessary [76].

- Catalyst loading

One of the key factors affecting photodegradation efficiency and overall cost is the amount of catalyst used. Increasing the TiO_2_ load does not only increase the number of active sites for pollutant adsorption, but it also increases the number of e−/h+ pairs, which speeds up the reaction rate [18,19,75]. However, if the amount of TiO_2_ is increased above a saturation level, the catalyst may agglomerate, resulting in a light screening deficiency [69,75]. This makes the excess TiO_2_ particles reduce the active surface area of the TiO_2_ exposed to illumination and consequently reduces the photocatalytic efficiency. Thus, the absence or obstruction of the irradiated light prevents it from penetrating the effluent’s interior mass, hence hindering photocatalysis [69]. Therefore, in order to avoid excessive usage and gain optimal photonic efficiency, an optimal value of catalyst dosage must be used [76].

- Light intensity

The light intensity ensures that sufficient photons of energy are delivered to the TiO_2_ surface-active sites. This should be significant enough to achieve a reasonable photocatalytic reaction rate, particularly in water treatment [51,69]. This is usually limited to photons with wavelengths that are smaller than the absorption edge of nearly 400 nm [51]. However, attenuation of radiation reaching the photocatalyst is one of the reasons for lower efficacy. Attenuation can occur for a variety of reasons, including insufficient light spatial distribution within the reactor, reflection losses, transmission losses, scattering, and so on [18,25]. According to Naldoni et al. [51], the rate of reaction can be associated with the high thermodynamic efficiency of reactor photons distributed within it.

- Catalyst type and temperature

The development of visible/solar light-active photocatalysts has been a continued effort in recent years [19,25,77,78]. Some of these photocatalysts have been synthesized and their efficacy in wastewater treatment has been determined. Additionally, most of the early photocatalysts developed were UV-only active and, therefore, research into photocatalysts’ physical and chemical properties is required to support their future prospective use. Modification of TiO_2_ with promising results has been reported as a solution. Some researchers have worked on incorporating activated carbon, graphite, graphene oxide, nanosheets, and other elements into TiO_2_ while others have focused on the incorporation of various metallic and nonmetallic additives [18,19,25,51,77,79,80]. Furthermore, doping metals and nonmetals into conventional photocatalysts enhances photoreactivity, and thus quantum efficiency, by altering the intrinsic and surface properties in a favorable approach [51]. In photocatalytic activity, temperature seems to have no effect. The ideal temperature range initiated by photon adsorption reaction is reported to be 20 to 80 °C [25]. Meanwhile, the activation energy, being relatively stable at these temperatures, makes it possible for a wide range of photon-induced reactions. However, an increase in temperature above 80 °C has been reported to have negative consequences on the overall photocatalytic process [25,51].

- Dissolved oxygen and contaminant concentration

The presence of dissolved oxygen (DO) is critical in TiO_2_ photocatalysis. The DO ensures that there are enough electron scavengers present to aid in the trapping of the excited conduction-band electrons during the recombination step [18,51,77]. The presence of DO has no effect on the adsorption onto the TiO_2_ catalyst surface since the reduction reaction occurs at several sites separate from where the oxidation occurs. The DO influences the formation of reactive oxygen species, the stabilization of radical intermediates, mineralization, and direct photocatalytic reactions [77]. The total amount of DO produced in a reactor is determined by a few factors such as the environmental condition, the catalyst type and concentration, the contaminant, and so on [80]. The pollutant’s initial concentration has a significant impact on its photodegradation. The effectiveness of photodegradation decreases as the initial concentration increases for two reasons: (i) equilibrium adsorption of the pollutant rises with increasing concentration and thus competes with adsorption of OH^−^ on the same site, and (ii) high-concentration pollutants absorb than the photocatalyst itself, which tends to prevent degradation [25].

## 3. Integrated Photocatalytic Membrane (IPM) Reactors

Membrane separation has become one of the most improved technologies for water treatment in recent decades due to its small carbon footprint, high separation efficiency, and ease of operation [2,39,71]. Membrane fouling is caused by the formation of a cake layer on the membrane surface and results in pore blocking in conventional membrane processes [81]. As a result, water flux is reduced significantly, and energy consumption and treatment costs are increased. Furthermore, membrane filtration can only concentrate contaminants into a high concentration retentate, which requires additional treatment prior to discharge [39,81,82]. Therefore, advancements in membrane science and technology are useful in developing a cost-effective membrane process. In essence, membrane technology has progressed to the point where there is a plethora of membranes made for specific pollutants as well as membrane configurations and technologies tailored for specific industries (milk production, beer production, desalination, solvent separation) and material regeneration [2,4,18,25,83].

Moreover, membranes are classified according to their pore sizes as microfiltration (MF), ultrafiltration (UF), nanofiltration (NF), and reverse osmosis (RO), which determine their selectivity [25,82,83]. Nanoporous membranes (0.1–1 nm) are made up of a thin film through which molecules are transported via solution diffusion. The driving forces across such membrane transport include pressure, concentration, or potential gradient [2,25,61]. Reverse osmosis (RO), NF and most recently forward osmosis (FO) are processes that use nonporous membranes. Microfiltration (MF) and ultrafiltration (UF) are two pressure-driven processes that use microporous membranes. MF membranes can separate particles with diameters ranging from 0.1 to 10 µm, while UF membranes can remove particles with diameters ranging from 1 to 100 nm [2,84].

Generally, to meet strict wastewater discharge standards, a conventional activated sludge process is used to biologically treat wastewater for organic/nutrient removal, accompanied by an MF/UF process to produce high-quality permeate water (i.e., removing particles and bacteria/viruses, etc.) Furthermore, membrane filtration can only trap contaminants in a high concentration retentate, which requires additional post-treatment prior to discharge. In contrast, photocatalytic membrane processes may degrade contaminants in feed solutions by producing oxygen-reactive radicals in the presence of UV light, preventing the formation of a cake layer on the membrane surface [3,45]. As a result, pore blocking is reduced, pollutant concentrations in the filtrate are reduced, and permeate quality is improved [3,45]. This section delves into the different types of membranes used as supports as well as related photocatalytic membrane types, preparation, attributes and applications of TiO_2_ photocatalytic membranes in removing contaminants in wastewater settings.

### 3.1. Types of IPM Reactors

Simple photocatalytic reactors that use TiO_2_-coated glass beads and a UV lamp to illuminate and activate sites are referred to as slurry/immobilized photocatalytic reactors or fixed-bed photocatalytic reactors [16,48,82]. In recent years, photocatalytic reactors have been upgraded in a variety of ways to increase the irradiated surface area and boost performance. Consistently, the difficulty in separating nano sized TiO_2_ particles from treated water has been attributed to its restricted use. To solve the separation problem, some researchers have attempted to salvage nano sized TiO_2_ particles from suspensions using membrane filtration [20,39,48,63,82,85].

**Figure 4:**
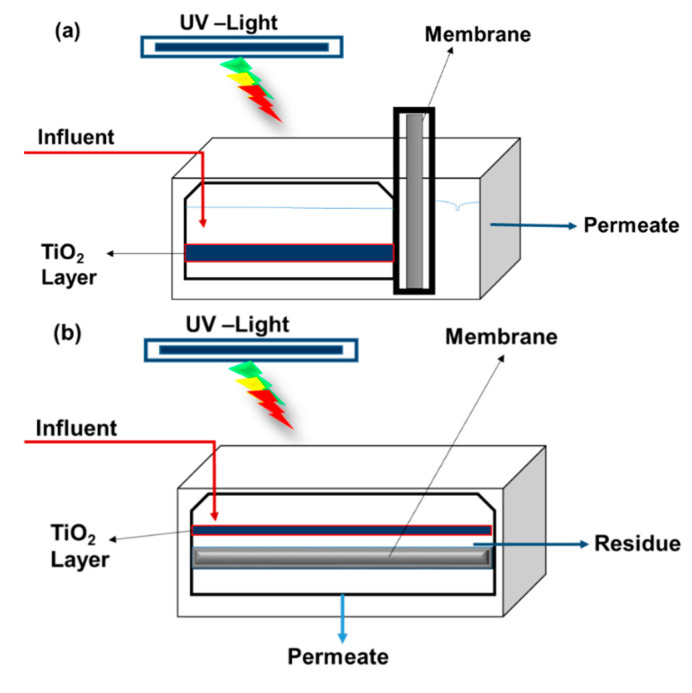

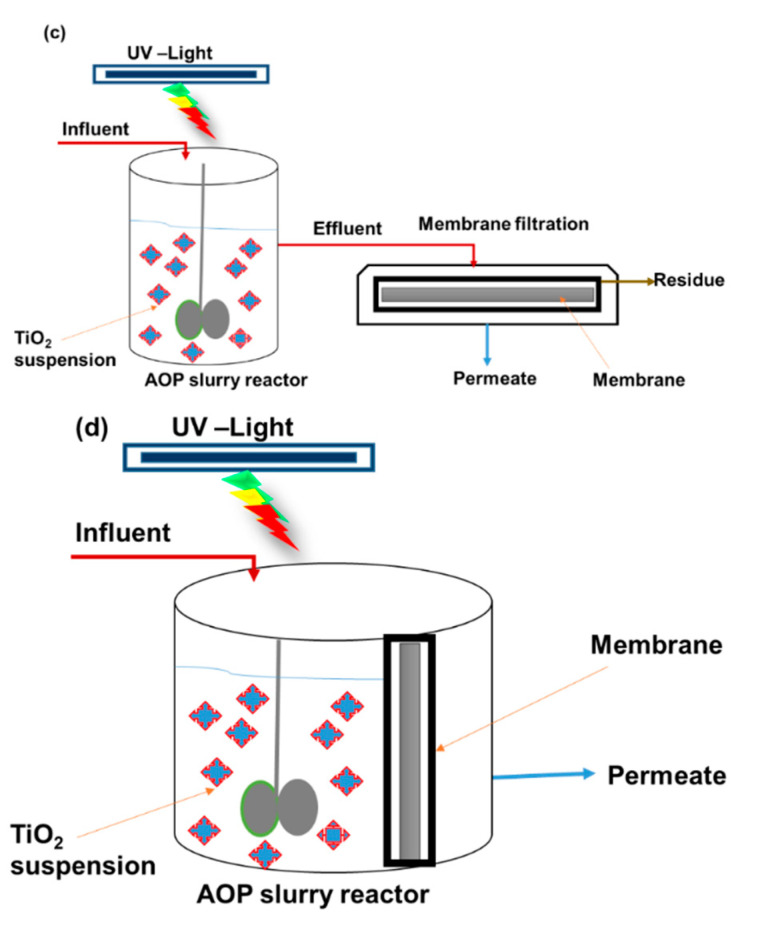
Schematic diagram of TiO_2_-based membrane reactor types: (**a**) a membrane inserted inside a photoreactor with internal walls coated with a photocatalyst such as TiO_2_, (**b**) a pure TiO_2_ porous photocatalytic membrane, (**c**) a slurry photocatalytic reactor followed by a membrane filtration unit, and (**d**) an inorganic or polymeric membrane submerged in a slurry photocatalytic reactor; adapted from [18,26,32].

IPMs are classified into four different configurations: (Figure 4a) a membrane inserted inside a photoreactor with internal walls coated with a photocatalyst such as TiO_2_; (Figure 4b) a pure TiO_2_ porous photocatalytic membrane; (Figure 4c) a slurry photocatalytic reactor followed by a membrane filtration unit; (Figure 4d) an inorganic or polymeric membrane submerged in a slurry photocatalytic reactor.

Among these four configurations, the photocatalytic membrane (Figure 4b) has potential advantages over the other three configurations [26,53]. Here, both the separation membrane and the TiO_2_ photocatalytic degradation of the organics occurs in a single unit [26]. In addition, photocatalytic membranes generally outperform conventional membranes in terms of reducing membrane fouling and improving permeate quality [85,86]. Even though the reactor configuration covers a broad range, the reactor design should ensure that the catalyst is irradiated uniformly [25]. This is because the photocatalytic surface available for the possible solutions can decrease and cause a loss in the photocatalytic efficiency.

Notwithstanding, IPMs have proven to be very promising in wastewater treatment settings, however, they are still limited in a large-scale application. In response, several organic, inorganic, metallic, and polymeric materials have been used as support materials in the fabrication of TiO_2_-based photocatalytic membrane reactors [25,26,53,70,87,88]. In contrast to immobilized TiO_2_ membranes, TiO_2_ nanofibers, nanowires, and nanotubes are also used to make pure TiO_2_ photocatalytic membranes. These are classified into two major categories: (i) freestanding pure TiO_2_ photocatalytic membrane reactors and (ii) composite TiO_2_ photocatalytic membrane reactors [87,89,90].

### 3.2. Freestanding Pure TiO_2_-Based Membrane Reactors

Engineered TiO_2_ nanoparticles have various properties that allow them to bind specific pollutants or catalyze degradation reactions when deposited or embedded on membrane surfaces [26,88,90]. However, the nanomaterial loading, matrix content, particle dispersion, nanoscale phase size, shape and orientation, as well as interactions between the phases all affect the hybrid material’s overall properties [26,90]. This has inspired interest in making TiO_2_-engineered membranes for a variety of applications, including self-cleaning, antimicrobial thin film coatings, photocatalysis, gas sensing and dye-sensitized solar cell nanofibers with well-controlled morphology [25,26,87,88,89,90]. One-dimensional freestanding TiO_2_ membrane reactors with various TiO_2_ morphologies, such as nanotubes (NTs), nanofibers (NFs), and nanowires (NWs), have been developed to improve photocatalytic activity and reduce membrane fouling [31,45].

#### 3.2.1. TiO_2_ Nanotube-Based Reactors

The photocatalyst surface-to-volume ratio of synthesized TiO_2_ NTs make them more advantageous for wastewater treatment. Some reported applications are presented in Table 3.

**Figure 5:**
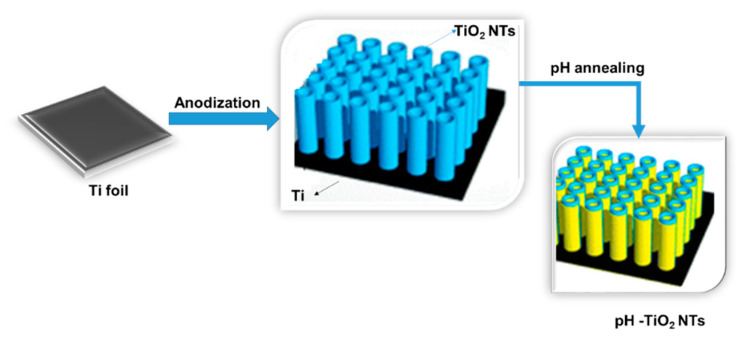
Schematic modification of TiO_2_ nanotubes; adapted from [83,92].

Although TiO_2_ NTs are available in both crystalline and amorphous forms to enhance performance, high-temperature annealing is required to convert the amorphous form to crystalline [83,91]. For example (Figure 5), from a two-electrode electrochemical cell with titanium foil as the working electrode, a freestanding TiO_2_ NT was synthesized using an anodization method.

In the preparation of TiO_2_ NTs, Cai et al. [83] stated that voltage pulses were applied for a short time at the end of the anodization process to achieve open nanochannels; this was a quick and successful bottom-opening method that did not involve the use of engraving solutions. Another method for creating a TiO_2_ nanotube alumina membrane was represented by Wang et al. [89], where the TiO_2_ NTs were developed by immersing the alumina membrane in a 2.2 pH titanium tetrafluoride (TiF_4_) aqueous solution. Their reported SEM image results showed that the TiO_2_ NTs were grafted and lined inside the channels of the alumina membrane. Chong et al. [84] reported that the effect of nanotube packing and grafting was highly dependent on the pH of the solution. Additionally, grafting time can be used to monitor TiO_2_ NT inner diameter, with a longer grafting time resulting in a smaller internal diameter and therefore, a lower pure water flux [91,92].

#### 3.2.2. TiO_2_ Nanofiber-Based Membranes

**Figure 6:**
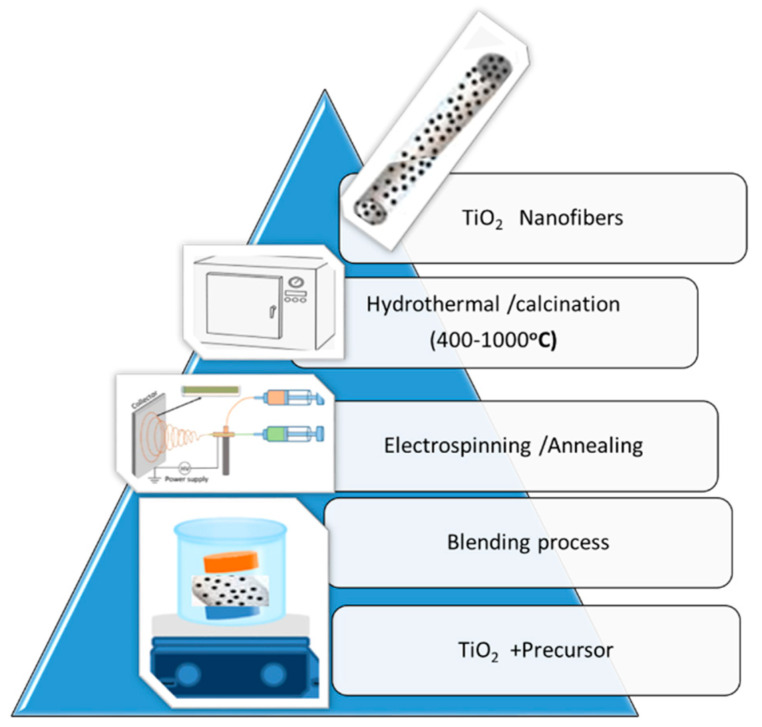
Schematic modification of TiO_2_ nanofibers using electropinning and hydrothermal techniques; adapted from [98,100].

Many methods of synthesis of TiO_2_ nanofibers (NF) have been developed, including sol–gel, hydrothermal, and electrospinning techniques (Figure 6).

Electrospinning is a versatile and efficient technique for synthesizing uniform fibers with a broad specific surface region [28,89,93]. In electrospinning, a polymer solution or melt containing a precursor salt of metal oxide in a syringe is subjected to a high static voltage [94,95]. The properties of nanofibers can be tweaked to provide more versatility in the end-product’s surface functionalities. Many studies use Ti-alkoxides and Ti-halides as TiO_2_ precursors, but these are insoluble in water [65,89,94,95,96,97,98]. TiO_2_ nanofibers made from water-soluble precursors make it possible to combine TiO_2_ with other metal oxides via water-soluble precursors [99]. At a voltage of 30 kV, nanofibers are electrodeposited, collected and dried at a temperature of 25 °C. Liu et al. [100] fabricated silver (Ag) nanoparticle-decorated TiO_2_ nanofiber (TiO_2_/Ag NF) membranes using the polyol synthesis mechanism. The Ag-decorated membrane (TiO_2_/Ag/CdS/Ag NF) was obtained by immersing TiO_2_/Ag/CdS nanofibers in an algae solution for 72 h [98,100].

#### 3.2.3. TiO_2_ Nanowires

**Figure 7:**
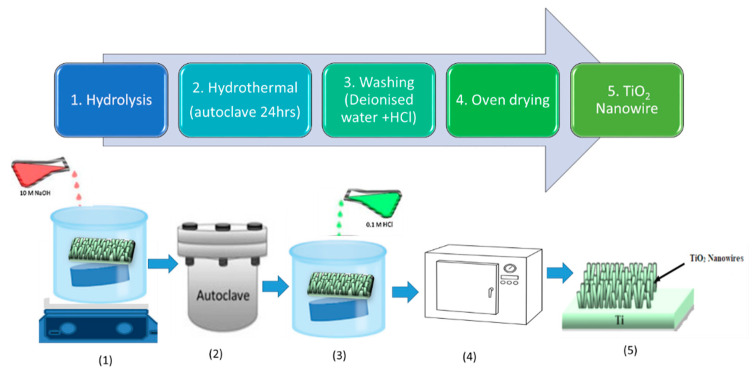
Schematic modification of TiO_2_ nanowire using hydrothermal techniques. (**1**) Hydrolysis, (**2**) Hydrothermal, (**3**) Washing, (**4**) Oven drying and (**5**) TiO_2_ nanowire.

The successful synthesis of titanium dioxide nanowires (NWs) was achieved using a novel approach based on a hydrothermal process [101,102]. A novel TiO_2_ nanowire nanostructure (Figure 7) was developed by hydrothermally treating TiO_2_ nanopowder as a precursor for 6 h with highly concentrated sodium hydroxide [102]. In the thermal evaporation processes reported in the literature, the sputtering of a Ti buffer layer and the evaporation of Ti at high temperatures are considered mandatory [102,103,104]. These processes require a lot of energy and a sophisticated setup for the nanostructures to expand [101]. The thermal oxidation method has been used to produce TiO_2_ NWs in recent studies [102,103]. The development of TiO_2_ NWs with a stable rutile phase is possible with this method. Zhang et al. [102] demonstrated that a direct oxidation of a Ti foil in an organic atmosphere was possible to develop a membrane filtration process and photocatalytic degradation of humic acid in wastewater.

### 3.3. Composite TiO_2_ Photocatalytic Membrane Reactors

TiO_2_ composite membrane reactors are classified based on the types of support materials used on the membrane. Among these are TiO_2_ polymer composite membranes, TiO_2_ ceramic composite membranes and TiO_2_-inorganic/organic materials composite membranes [82,105]. The TiO_2_ NPs can either be deposited onto the membrane surface or dispersed in the polymer dope solution prior to membrane casting.

#### 3.3.1. TiO_2_ Nanoparticle-Coated Polymer Membranes

In photocatalytic membranes, nanoscale inorganic/organic photocatalysts are embedded in a membrane matrix to improve the properties of the resulting polymer [82,105,106,107]. Hamed et al. [108] eluded that TiO_2_ nanoparticles can coordinate with the surface of the polymer membrane and Ti^+4^ makes ion connections with oxygen atoms of carboxylic groups of polymers or by making H-bonding with carbonyl or hydroxyl groups on the TiO_2_ surface. Hyeok et al. [109] increased the hydrophilicity of a commercial membrane via plasma immersion in a nitrogen-purged aqueous acrylic acid solution at various concentrations. The plasma treatment irradiated the membrane surface, causing radicals to form and promoting grafting reactions. TiO_2_ was then successfully deposited onto the functionalized membrane surface [82,105].

Some researchers have investigated the photocatalytic behavior of TiO_2_ nanoparticles photografted onto commercial polymer membranes, such as polyester membranes [24,82,108,110]. Dong et al. [98] used the activation reaction to coat TiO_2_ nanotubes on a polyurethane (PU) membrane surface. The TiO_2_ nanotube suspension was formed by ultrasonically dissolving salinized TiO_2_ nanotubes in toluene for 10 min. The activated PU membrane was added to the coating process, which was then heated to 60 °C. Furthermore, the TiO_2_ nanotube–PU hybrid membrane was collected and washed in ethanol before being dried in a vacuum oven. When the membranes were modified with diethanolamine (DEA), Hamed et al. [108] found that a high concentration of TiO_2_ coated on the polyvinylidene fluoride (PVDF) membrane improved pure water flux. Although embedding nanoparticles onto membranes remove unwanted nanoparticles after treatment, the polymer phase material covering the nanoparticles can reduce the active usable surface area [107]. Owing to the blockage of the membrane pores by TiO_2_ nanoparticles, the photocatalytic membrane’s pure water flux usually decreases as the concentration of TiO_2_ in the coating phase rises [58,107,109,111].

#### 3.3.2. TiO_2_-Based Polymeric Membranes

Polymer membranes are one of the most popular membranes used in wastewater treatment and water purification (Table 4).

Polymers reported as support membranes of photocatalysts include polyamide, polyvinylidene fluoride (PVDF) [23], polyethersulfone (PES), poly (vinylidene fluoride) (PVDF) [24], sulfonated polyethersulfone (SPES), poly (vinylidene fluoride) [105,106,107,108], polyurethane (PU) [98], polyethylene terephthalate (PET) [82], polyester [58], polyacrylonitrile (PAN) [43] and polytetrafluoroethylene (PTFE) [109,110,111,112,113,114]. Polymer–inorganic nanoparticle composite membranes can be divided into two groups based on their structure: (a) polymer and inorganic phases linked by covalent bonds, and (b) polymer and inorganic phases linked by van der Waals forces or hydrogen bonds [113]. The TiO_2_ nanoparticles coalesce with the surface of the polymer membrane during the membrane preparation process. Likewise, Ti^+4^ makes ion connections with oxygen atoms of carboxylic groups of polymers or by making H-bonds with carbonyl or hydroxyl groups on TiO_2_ surfaces [107,111].

#### 3.3.3. TiO_2_ Ceramic Membranes

Ceramic membranes, especially Al_2_O_3_ membranes, have been extensively investigated as supports for photocatalyst immobilization in membrane photocatalytic processes (Table 5).

These are more chemically, thermally and mechanically stable than polymeric membranes. A summary of comparing the ceramic and polymeric membranes is presented in Table 6.

Polymeric membranes are commonly used for the treatment of industrial wastewater due to their availability [115]. On the other hand, ceramic membranes have an advantage over other wastewater treatment options because of their chemical and thermal stability [18,20,115]. Ceramic membranes, for example, can withstand high chlorine doses and work between pH levels of 1 to 14 and temperatures of up to 500 °C. Despite the advantages of ceramic membranes over polymeric membranes, their use has been restricted due to their high initial cost [20].

### 3.4. TiO_2_-Based Membrane Modification Techniques

TiO_2_-based catalysts can be deposited on structured membranes through aqueous or gaseous routes [117]. Membranes with desired morphology and properties are prepared using sol–gel, immersion precipitation, vapor-mediated phase separation and immersion precipitation, electrospinning, dip-coating, sintering, track-etching, stretching, template-leaching, and phase-inversion techniques [71,86,87,90,108,112,113]. Aqueous or gaseous routes can also be used to deposit TiO_2_-based catalysts on standardized membranes. The advantages and drawbacks of various methods for immobilizing TiO_2_ catalysts on membranes are summarized in Table 7.

Among them, the sol–gel method is the most used method for making TiO_2_ photocatalysts and subsequent incorporation of membrane matrices. This method is notable for producing unique stable structures at low temperatures as well as for its excellent chemical homogeneity [85,108,113]. Controlling the precursor chemistry and processing conditions will tailor the compositional and microstructural properties of nano-sized samples. As precursors, inorganic metal salts such as titanyl sulphate, titanium tetrachloride and others (non-alkoxide), and metal alkoxides such as titanium (IV) butoxide are commonly used [90,118]. The pH of the reaction medium, the water/alkoxide ratio, and reaction temperatures can influence the sol–gel phase. Horovitz et al. [119] prepared a TiO_2_ sol by stirring an ethanol solution in an ice bath while adding a specific amount of TiO_2_ precursor. The second solution was gradually added with a 50:1:3 volume ratio of ethanol, water, and acetic acid. The mixed solution was held at 20 °C for 30 min before being ultrasonicated to produce a visible TiO_2_ sol. Dip-coating was achieved by immersing a substrate membrane in the sol for a set period and then drying the coated membrane [71,86]. Penboon et al. [85] made a distinction between TiO_2_-coated membranes and TiO_2_-blended polymer membranes. It was deduced that the TiO_2_-blended membranes had a more porous structure, but the coated TiO_2_ composite membranes had greater antifouling properties and long-term flux stability. They went on to say that TiO_2_ nanoparticles, which were spread on the membrane’s surface using the coating process, could minimize pore blockage. Furthermore, TiO_2_-coated membranes are more hydrophilic than TiO_2_-blended membranes.

### 3.5. Characteristics of Photocatalytic Membranes

Characterization of photocatalytic membranes is based on two parameters, membrane morphology and efficiency (permeability, flux, rejection, and separation factors), which are used to describe the membranes (pore size, pore size distribution, thickness of membrane, charge density and pore shape, chemical properties, and physical properties) [3,18,120]. Improving the hydrophilicity of the membrane surface for TiO_2_ nanoparticle deposition involves the introduction of functional groups to the membrane matrix (Table 8).

On the other hand, little research has been done on the impact of immersion time on the properties of photocatalytic membranes. According to Zhang et al. [121], longer immersion periods resulted in more TiO_2_ nanoparticles coating the membrane surface, which appeared to clog the membrane pores and reduce flux. Nanomaterials and membranes must be characterized in order to understand their structure, chemistry, morphology, surface charge, wet potential, tensile strength and selectivity for a particular application [39,71,82,112]. Notwithstanding, membrane efficiency degrades over time due to a variety of factors such as fouling, chemical contamination, disintegration and cleaning. Therefore, characterization in this case using particular techniques provides useful information about membrane structure and topography in order to gain insight into performance loss or affected membrane structure.

Similarly, modified membranes (modification with nanomaterials) are also characterized in order to better understand the modified surface layer and how it affects membrane performance. Commonly used techniques and methods for characterization include scanning electron microscopy (SEM), transmission electron microscopy (TEM), Fourier transform infrared spectroscopy (FTIR), X-ray diffraction (XRD), X-ray photo electron spectroscopy (XPS), thermogravimetric analysis (TGA), energy dispersive X-ray spectroscopy (EDX), dynamic light scattering (DLS), contact angle, porosity, and zeta potential (Table 8). Thiruvenkatachari et al. [19] reported that after 30 min of reverse osmosis-pressurized activity, some TiO_2_ particles detached from the membranes, but no further loss occurred after 7 days that was evidently noticeable in SEM images. Koseoglu-Imer and Koyuncu [105] eluded that diethanolamine increased the hydroxyl groups on the membrane surface, lowering the steric hindrance for attracting TiO_2_ nanoparticles. The content of TiO_2_ on the DEA-modified membrane was three times higher than on the membrane without DEA, according to the results of energy disperse X-ray spectroscopy.

Furthermore, membrane fouling is the accumulation of macromolecules (polysaccharides, proteins), colloids, microorganisms (bacteria, viruses) and salts (multivalent/monovalent) on the surface or within the pores of a membrane [45,113]. Fouling has a few significant drawbacks in membrane technology, including a reduction in permeation flux, a change in selectivity, and a reduction in membrane life during filtration. Membrane fouling is categorized into inorganic fouling, colloidal fouling, organic fouling and biofouling [45,113,118,122]. Therefore, developing photocatalytic membranes (fouling resistant membranes) are a safer alternative for fouling mitigation since they reduce foulant coagulation on the membrane surface, which contributes to clogging.

### 3.6. Application of TiO_2_-Based Membranes

Photocatalytic membranes, which combine membrane filtration with photocatalytic degradation of organics and subsequent antibacterial treatment in a single system, have shown great prospects in water and wastewater treatment settings [12,123,124,125,126,127]. Furthermore, membrane flux is a critical parameter for the photocatalytic membrane system, as it can be enhanced by multilayered deposition of TiO_2_ nanoparticles on the membrane surface, but pore blockage can minimize water flux in some cases [20,87]. In order to achieve the best overall performance of the membrane, the optimal balance between photocatalytic activity and membrane flux must be considered.

#### 3.6.1. Wastewater Treatment

Photocatalytic membranes are becoming more prominent to deal with emerging contaminants (antibiotics, pharmaceuticals, nanoplastics, and so on) re-routed to wastewater treatment plants, as well as pre- and post-treatment concerns [128,129,130]. Increased attempts to eliminate toxic contaminants and pathogens from water have come about from the need for high-quality drinking water [12,15]. Photocatalytic membranes have proven to be more effective in removing recalcitrant organic matter than conventional wastewater treatment processes such as coagulation and filtration [131]. Therefore, they have been the subject of comprehensive research over the last few decades for the removal of toxic organic and inorganic compounds from water (Table 9).

Zheng [20] reported on photocatalytic membranes for the removal of heavy metals such as mercury, lead, mercury, and cadmium from wastewater settings. Other researchers have reported that multilayered nanocomposite membranes have promising properties for heavy metal ion adsorption, photocatalysis, self-cleaning, enhanced dye rejection performance and reduced fouling [39,61,81,82]. Batch reactors and photocatalytic polycrystalline TiO_2_ membrane reactors were used by Shetty et al. [131] to degrade various pharmaceuticals from water at various pH levels. Due to the catalysts’ different hydrophilicity/hydrophobicity at different pH levels, the substrates showed different adsorption on catalyst surfaces when the system was in recirculation mode. The treated water had a permeate flux of 31.5–60.0 L/h.m^2^ in both the alkaline and acidic mediums, with a rejection percentage of 10–60% for furosemide and 5–30% for ranitidine [131]. Table 9 summarizes the removal of pollutants using different types of photocatalytic membranes.

#### 3.6.2. Other Industrial Applications

The development and production of simple and responsive biosensors have sparked a lot of interest due to their wide range of applications, including disease detection, drug discovery, food protection and environmental monitoring [82,85,106,133,134]. Due to the small active surface area of microelectrodes and the low recognition signal, electrochemical biosensors have a difficult time working [125]. As a result, chemical and thermal stability with good biocompatibility makes TiO_2_ appear to be a promising biosensor material [48]. Therefore, combining it with membranes would improve its suitability for environmentally friendly designs and applications. Examples of these materials include TiO_2_ nanotubes, nanosheets, solgel matrices, and three-dimensional microporous matrices [48,71,98,125]. Chen and Zhao [39] reported their first study on the production of a Fe_3_O_4_@TiO_2_ magnetic NP-based disposable test strip immunosensor that uses a multienzyme labeled amplification strategy to detect organophosphorus (OP) pesticides in human plasma.

## 4. Challenges and Future Prospects of Photocatalytic Membranes

Membrane-based technologies are gaining worldwide popularity in wastewater treatment settings due to their high separation efficiencies, low cost, small carbon footprint and ease of operation. This process can also be used to eliminate a wide variety of harmful pollutants, including pesticides and herbicides used in agriculture, as well as dyes and toxic metal ions used in industry. Despite extensive research and thorough studies of photocatalytic semiconductors (TiO_2_), many aspects of this process remain unsolved. One of the pressing issues as to whether the photocatalytic process should be used as a pretreatment or as a standalone form of water purification is the operating conditions that pose major constraints. Consequentially, the chemical composition and pH of industrial wastewater vary by region. Therefore, efforts should be made to develop photocatalytic membrane materials that can be used under a wide range or optimized operating conditions such as temperature, pH and contaminant concentrations. Furthermore, to achieve continuous irradiation with visible light over a wider range of operating conditions, a significant amount of research should be devoted to the doping and modification of TiO_2_ and other semiconductors. However, catalyst immobilization is an obstacle that must be overcome in order to maximize the photocatalysts’ irradiated surface area. This is needed to avoid issues such as catalyst recovery and agglomeration, which are very common in slurry-based photoreactors.

### 4.1. Photocatalytic Membranes

Membrane fouling, which is caused by the accumulation of anthropogenic organic contaminants and microorganisms on the membrane pores’ surface, results in poor water quality, high operating costs, and a limited membrane life. In order to perform equal photocatalytic operation with minimal water flow consequences, an adequate level of photocatalysts should be incorporated into the membrane. In addition to TiO_2_ loading, light intensity and irradiation time influence membrane quality.

In order to execute reasonable photocatalytic activity with minimal water flow repercussions, an appropriate level of the photocatalyst should be incorporated into the membrane. Membrane performance is also influenced by light intensity and irradiation time in addition to TiO_2_ loading. Addressing this, photocatalytic membranes have been reported to have better fouling resistance as well as antimicrobial properties. Two-dimensional nanosheets with graphene have been reported to be useful for membrane applications. Additionally, combinations of nanowires with graphene sheets have also gained attention recently. Such types of structures provide antifouling and antimicrobial properties as well as hydrophilicity and aqueous stability.

### 4.2. Magnetized TiO_2_ Photocatalytic Membranes

In order to implement heterogeneous photocatalysis into practical water and wastewater treatment applications, the overall cost of the process should be minimized. One of the ways to decrease the cost is to improve the recyclability of the photocatalyst. This can be achieved by introducing magnetic constituents into the photocatalytic nanocomposite to enhance recoverability by the application of an external magnetic field. Magnetic components (Fe_3_O_4_) can benefit in the recovery of photocatalyst particles by preventing them from clumping together [39,67]. Magnetite (Fe_3_O_4_) NPs, maghemite (Fe_2_O_3_) NPs and nanorods, and hexagonal ferrites are examples of ferritic nanomaterials that are simple to recover by magnetic fields and are re-usable due to their long-term stability.

In some studies, Fe_3_O_4_ and TiO_2_ nanoparticles have also been successfully used as a filler for composite membranes. This opens a broad spectrum of recycling possibilities in the form of magnetized photocatalytic membrane applications (Table 10).

Thus, Fe_3_O_4_ NPs display exceptional superparamagnetic behavior, which provides an additional benefit of a much easier magnetic separation of the semiconductor and the solution. The coprecipitation method or thermal decomposition method is widely used to make Fe_3_O_4_ nanoparticles, with minor variations such as room temperature (25–80 °C). The exemplary adsorption potential for toxic metal ion removal was achieved by the finite-size effect of magnetic nanoparticles with high surface-to-volume ratios [118]. Magnetite has also been used as an additive due to its unique features, such as physicochemical properties and high biocompatibility. Many polymeric-based membranes, such as polysulfide and polyether sulfone, had their membranes magnetized with magnetite to strengthen antifouling properties. However, knowledge of magnetized TiO_2_ photocatalytic membranes is still limited and therefore requires more attention.

### 4.3. Life Cycle Assessment (LCA)

A life cycle assessment (LCA) is needed to incorporate TiO_2_ photocatalytic-based membrane technology on a large scale [3,84]. LCA is one of the most useful instruments for determining a process’s environmental effects as well as its viability and costs. Although there is a large amount of information in the literature about the recyclability and reusability of TiO_2_ photocatalysts, there is still limited knowledge about their end of life [3,19]. The photocatalytic process performance, on the other hand, is highly dependent on the feed water’s consistency, the operating conditions and type of treatment configuration. Apart from the environmental impacts of high organic wastewater, the amount of energy required has a major impact on the photocatalysis process [3,84]. This is one of the reasons why solar photocatalysis has received so much attention in recent years. Consequently, the toxicological and environmental impacts of a wastewater treatment facility can be detected and minimized using LCA.

## 5. Conclusions

This review study presents the photocatalytic membrane process as a game-changing technology to mitigate fouling and other drawbacks of membrane processes in addressing water scarcity and major environmental challenges. Characteristics of TiO_2_-based photocatalytic membranes with insight into their types, fabrication techniques, operational conditions and applications in water settings are discussed. As a result, the advancement of photocatalytic membrane reactors based on a wide range of nanomaterials and TiO_2_ photocatalyst phase modification is anticipated to have an unparalleled future in water and wastewater treatment settings. The development of magnetite TiO_2_ photocatalytic membranes was found to be an ecofriendly choice to increase photocatalytic and recoverability activities as well as reduce membrane fouling. Additionally, incorporating magnetized TiO_2_ photocatalytic membranes as a post-treatment to an existing biological system can reduce eutrophication potential while increasing biomethane potential for energy usage. The prospects of life cycle assessment, research and development of magnetic photoactive-based membranes is foreseen to be economically viable with the possible potential to resolve the current membrane setbacks and bring the application of this technology to industrial scale.

## Figures and Tables

**Table 1:** Types of advanced oxidation processes; adapted from [19,46].

Chemical Process	Photochemical Process
Wet air oxidation	Photo-Fenton reaction
Supercritical water oxidation	UV/ultrasound system
Ultrasound/H_2_O_2_ system	UV/O_3_/H_2_O_2_ system
Ultrasound/Sonolysis	UV/H_2_O_2_ system
Fenton reaction	UV/O_3_ system
Ozonation in alkaline	UV photolysis
O_3_/H_2_O_2_ system	UV/O_2_/TiO_2_ system
Electron—Fenton reaction	UV/H_2_O_2_/TiO_2_ system

**Table 2:** Chemo-physical properties of TiO_2_ (adapted from [49,54]).

Crystalline Forms	Anatase	Rutile	Brookite
Crystalline structure	Tetragonal	Tetragonal	Rhombohedral
Lattice constants (nm)	a = b = 0.3733 c = 0.9370	a = b = 0.4584 c = 0.2953	a = 0.5436; b = 0.9166 c = 0.5135
Density (g.cm^−3^)	3.83	4.24	4.17
Bravais lattice	Simple, body-centered	Simple, body-centered	Simple
Melting point (°C)	Turning into rutile	1870	Turning into rutile
Boiling point (°C)	2927	-	-
Band gap (eV)	3.2	3.0	-
Refractive index (ng)	2.5688	2.9467	2.809
Standard heat capacity (cp)	55.52	55.60	-
Dielectric constant	55	110–117	78

**Table 3:** Removal of pollutants using TiO_2_ nanotubes.

TiO_2_ Nanotubes	Pollutant	Removal Efficiency (%)	Reference
TiO_2_ arrays	Acid orange 7	77	[91]
TiO_2_ –Au/Ag	Acid orange 7	85	[91]
TiO_2_	Acid orange 7	99	[91]
TiO_2_	Oil	99	[92]
TiO_2_/CdS	Rhodamine B	60	[75]
Ag–TiO_2_/HPA/Al_2_O_3_	Humic acid	88	[47]
TiO_2_	Humic acid	98	[47]
Si-doped TiO_2_	Reactive Red ED-28	40	[83]
TiO_2_	Methylene blue	68	[83]
Silica/TiO_2_	Direct black 168	85	[83]

**Table 4:** Polymer membranes with different TiO_2_ types.

Polymer Membrane	Types of TiO_2_	Deposition Time (min)	Reference
Polyethersulfone (PES) Ultrafiltration membrane	Degussa 25	15–30 60	[112]
Poly (vinylidene fluoride) Plasma-grafted poly acrylic acid (PAA)	TiO_2_ suspension	30	[113]
Polyamide thin film supported on polysulfide	TiO_2_ colloidal solution	60	[107]
Polyether sulfone (PES) Polyimide blend membrane (PI)	Degussa 25	15	[109]

**Table 5:** Synthesized TiO_2_ ceramic membranes.

Type of Membrane	Type of TiO_2_ Precursor	Calcination Conditions	Reference
Al_2_O_3_	Tetra-n-butyl titanate	400 °C for 6 h by 100 °C/h	[18]
Al_2_O_3_	Tetraethyl orthosilicate	400 °C for 2 h by 100 °C/h	
ZrO_2_	Tetrabutyl orthotitanate	530 °C for 1 h by 100 °C/h	[20]
Al_2_O_3_	Tetra-n-butyl titanate	500 °C for 2 h by 2 °C/min	[115]
Al_2_O_3-_ZrO_2_	Tetraisopropoxide titanium	510 °C for 2 h by 5 °C/min	
Al_2_O_3_	Titanium tetraisopropoxide	500 °C for 15 min by 3 °C/min	[20]
Activated carbon filter	Tetra-n-butyl titanate	200 °C for 15h	[18]
Al_2_O_3_	Tetraisopropyl orthotitatnate	450 °C by 10 °C/h	[20]
Al_2_O_3_	Commercial TiO_2_	450 °C by 0.5/min	[115]

**Table 6:** Comparison of different types of membranes; adapted from [116].

Membrane Type	Polymeric Membrane		Ceramic Membrane
Membrane morphology	Hollow fiber	Flat sheet	Tubular membrane
Stability	Medium	Medium	Good
Price	Less expensive	Less expensive	Expensive
Configuration mode	Internal or external	Internal or external	Internal or external
Module processing	Quite simple	Easy	Hard

**Table 7:** Advantages and disadvantages of different methods used for immobilizing TiO_2_ photocatalysts [116,117].

Synthesis Technique Type	Advantage	Disadvantage
Chemical vapor deposition	Processing time is short. Suitable for high-deposition-rate uniform films.	High deposition temperature (>600 °C) is required.
		Due to varying evaporation times, deposition of many sources of precursors is difficult. When a vacuum system is used, the price skyrockets.
Physical vapor deposition	Low-to-medium deposition temperature (<600 °C) required. Does not involve complex chemical reactions.	Expensive as vacuum systems are used. Deposition of multiple sources of precursors are difficult.
Sol–gel	High purity of materials	Hydrolysis rate is difficult to control.
	Homogeneity is achievable.	
	Versatile means processing and control of parameters.	
	Large surface area materials are produced.	Longer processing time required.
	Chemical bonding results in strong adherence of coating to the substrate.	Calcination at higher temperatures is required.

**Table 8:** Modification and characterization techniques of TiO_2_-based membranes.

Membrane Types	Modification Methods	Characterization Methods	Reference
TiO_2_–Polysulfone (PS)	Blending	FTIR, SEM/EDS. XRD, contact angle, zeta potential	[105]
TiO_2_–Polyethersulfone (PES)	Sol–gel	FTIR, XRD, SEM/EDS	[118]
TiO_2_/PES	Phase inversion	Contact angle, FT-IR, TGA, pore size distribution, SEM	[123]
TiO_2_/Cellulose Acetate	Dip-coating	Contact angle, viscosity, SEM, FTIR	[113]
ZrO_2_ PVDF	Blending	SEM, TEM, AFM, XPS, TGA, contact angle	[95]
Cellulose acetate- Polyurethane (CA-PU)	Blending	SEM, TEM, AFM, XPS, TGA, contact angle	[19]
Polyvinylchloride (PVC)	Sol–gel	SEM, FTIR, DLS, TGA	[105]

**Table 9:** Application of TiO_2_-based membranes in wastewater settings.

Hybrid Membranes.	Pollutants	Removal Efficiency (%)	Reference
UV-ZrO_2_/TiO_2_–Al_2_O_3_	Methylene blue	95	[25]
Solar-Ag/TiO_2_	Methylene blue	80	[18]
UV-TiO_2_/AL_2_O_3_	Direct black 168	63	[97]
UV-ZrO_2_/TiO_2_–Al_2_O_3_	Methyl orange	95	[132]
UV-TiO_2_/PVDF	Reactive black 5	99	[51]
UV-TiO_2_–Al_2_O_3_	Reactive black 5	70	[97]
UV-TiO_2_ nanotube	Rhodamine B	70	[131]
TiO_2_ nanotube	Humic acid	28	[112]
UV-TiO_2_ nanowire	Humic acid	60	[102]
UV-TiO_2_/Al_2_O_3_	Humic acid	97	[112]
UV-TiO_2_ nanotube	Humic acid	95	[102]

**Table 10:** Examples of magnetite heterojunction photocatalyst performance.

Composition	Pollutant	Operating Condition	Efficiency (%)	Reference
Fe_3_O_4_ (0.075 gL^−1^)	*E. coli*	UV @ *t* = 60 min	50.5	[41]
Fe_3_O_4_ –TiO_2_	Bisphenol (10 ppm)	UV @ *t* = 60 min	92	[106]
Fe_3_O_4_–ZnO	Rhodamine B (7 ppm)	UV @ *t* = 60 min	99.3	[52]
Fe_3_O_4_–ZnO-rGO	Methylene violet (408 ppm)	UV vis @ *t* = 120 min	83.5	[52]
Bi_2_O_3_–Fe_3_O_4_	Ibuprofen (2.1 ppm)	UV vis @ *t* = 120 min	>95	[39]
Fe_3_O_4_–CuO–ZnO–nano graphene	Methylene blue (30 ppm)	UV @ *t* = 120 min	93	[67]
Fe_3_O_4_–ZnO–CoWO_4_	Rhodamine B (4.8 ppm)	UV vis @ *t* = 405 min	98.3	[134]
Fe_3_O_4_–Bi_2_O_3_	Ciprofloxacin	UV vis @ *t* = 240 min	98.3	[131]

## Data Availability

Not applicable.
